# Spatial patterns of the congenital heart disease prevalence among 0- to 14-year-old children in Sichuan Basin, P. R China, from 2004 to 2009

**DOI:** 10.1186/1471-2458-14-595

**Published:** 2014-06-12

**Authors:** Li-Guang Ma, Jun Zhao, Zhou-Peng Ren, Yuan-Yuan Wang, Zuo-Qi Peng, Jin-Feng Wang, Xu Ma

**Affiliations:** 1 National Research Institute for Family Planning, Beijing 100081, People's Republic of China; 2 State Key Laboratory of Resources and Environmental Information System, Institute of Geographical Sciences and Natural Resources Research, Chinese Academy of Sciences, Beijing 100101, People's Republic of China

**Keywords:** Congenital heart disease(CHD), Hierarchical Bayesian model(HB), Spatial autocorrelation, Hot-spot analysis, Sichuan Basin

## Abstract

**Background:**

Congenital heart disease (CHD) is the most common type of major birth defects in Sichuan, the most populous province in China. The detailed etiology of CHD is unknown but some environmental factors are suspected as the cause of this disease. However, the geographical variations in CHD prevalence would be highly valuable in providing a clue on the role of the environment in CHD etiology. Here, we investigate the spatial patterns and geographic differences in CHD prevalence among 0- to 14-year-old children, discuss the possible environmental risk factors that might be associated with CHD prevalence in Sichuan Basin from 2004 to 2009.

**Methods:**

The hierarchical Bayesian model was used to estimate CHD prevalence at the township level. Spatial autocorrelation statistics were performed, and a hot-spot analysis with different distance thresholds was used to identify the spatial pattern of CHD prevalence. Distribution and clustering maps were drawn using geographic information system tools.

**Results:**

CHD prevalence was significantly clustered in Sichuan Basin in different spatial scale. Typical hot/cold clusters were identified, and possible CHD causes were discussed. The association between selected hypothetical environmental factors of maternal exposure and CHD prevalence was evaluated.

**Conclusions:**

The largest hot-spot clustering phenomena and the CHD prevalence clustering trend among 0- to 14-year-old children in the study area showed a plausibly close similarity with those observed in the Tuojiang River Basin. The high ecological risk of heavy metal(Cd, As, and Pb)sediments in the middle and lower streams of the Tuojiang River watershed and ammonia–nitrogen pollution may have contribution to the high prevalence of CHD in this area.

## Background

Congenital heart disease(CHD) refers to a malformation of the cardiovascular system and accounts for nearly one-third of all major congenital anomalies [1]. Heart malformations are the most common form of birth defects, occurring in approximately 8 per 1000 live births [2]. Surveillance data shows that CHD has the highest prevalence among other birth defects in Sichuan Province in recent years [3-6]. The proportion of birth defects related to infant mortality has recently increased, and CHD is now the most common cause of infant mortality and the leading cause of disability in young children [7], thereby increasing healthcare costs each year [8,9].

The pathogenesis of CHD is complicated and its underlying mechanism remains unknown. A group of CHD lesions with unknown etiology follows a multifactorial inheritance model, approximately 90% CHD cases are multifactorial [10-12], which implicates both genetic and environmental factors in disease development. Approximately 80% CHD cases are multifactorial and arise through various combinations of genetic and environmental factors [1,13].

Environmental factors contribute to 10% birth defects, but most birth defects are presumed to be caused by the combination or interaction of genetic and environmental factors [14]. Epidemiological research has yet to focus on the demographic, familial, social, genetic, and ethnic factors associated with the prevalence of CHD.

From the spatial epidemiology perspective, significant geographic differences occur in CHD prevalence [1]. Some studies have focused on geographical variations in CHD prevalence [1,15-19], and CHD prevalence has been demonstrated to be closely related with elevation and latitude [20-22]. In addition, there is a clear and seasonal variation in CHD prevalence [23-27]. Maternal exposure to environmental factors such as ambient air pollution [28-33], heavy metals, and micronutrients are positively related to CHD prevalence because elements in the soil, water, and air affect human beings directly or indirectly [1,34-36]. The physical environment such as solar radiation and magnetic fields also have influence on CHD prevalence [37,38]. Furthermore, socio-economic and lifestyle habits affect CHD prevalence. However, the extent of the contribution of these factors to CHD prevalence in the study area is unknown.

The purpose of this study was to detect spatial patterns of CHD prevalence at various geographical scales and to explore the possible links between CHD and environmental changes.

## Methods

### Study design

In this study, we mapped the prevalence of CHD among 0- to 14-year-old children at the township level in our study area firstly. In order to eliminate the dependence of the sampling variance on population size and the CHD prevalence, the hierarchical Bayesian model(HB) was employed to address the problem of a small population during explorative mapping of prevalence and to stabilize local estimates of CHD prevalence. Subsequently, global Moran’s I statistic and local indicator of spatial association(LISA) statistic [39] were used to detect regions with high prevalence of CHD and the local Getis'sGi* method [40] was used to draw a prevalence map of CHD using the geographic information system(GIS). Finally, we try to explore the association between the high-prevalence clustering pattern of CHD and potential environmental risk factors.

All data analysis, including data processing, mapping, and spatial statistics were conducted using the ArcGIS and GeoDa 0.9.5-i software. The hierarchical Bayesian model and the publically available Winbugs 1.4 software [41] were used along with the Markov Chain Monte Carlo method.

### Study area

The study area is situated in the eastern part of Sichuan Province in southwestern China, located in the Sichuan Basin with distinct geographic environment. The study area includes 13 municipalities comprising of 105 counties and 685 townships, due to its relative flatness and fertile ground, it’s the most populous region in China with a population of near 70 million, and the population density is approximately 500–700 persons/km^2^.

Sichuan Basin is bordered by mountains and consists of low hills and alluvial plains with an elevation of 250–700 m. The Yangtze River passes through the southern part of the basin. Several major rivers such as the Minjiang River in central Sichuan and the Jialing River are tributaries of the upper Yangtze River.

**Figure 1:**
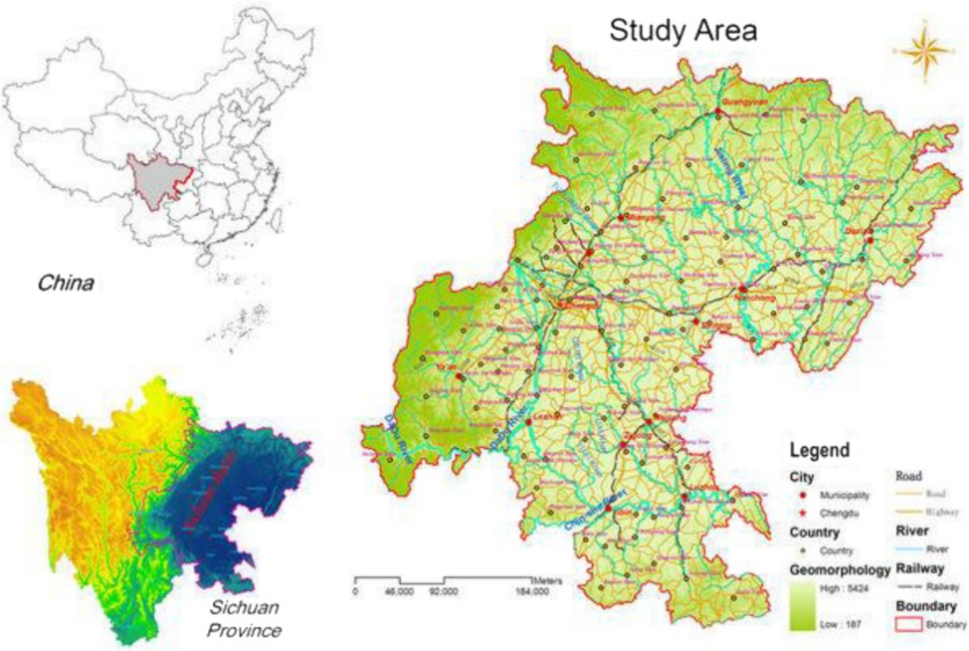
Study Area Location Map.

Due to the unique prominent geology, geomorphology, and climatic characteristics, the study area is mostly covered by farms and cities. The basin with high population density and the cultivated land in the basin account for 85% of the total cultivated land in Sichuan Province. The basin is the central distribution of Chinese Mesozoic continental red beds, with plentiful mineral resources, various land use types, and well-developed industries, which generates a peculiar basin environment [42]. A map of the study area with the major cities, highways, rivers, and township boundaries is shown in Figure 1.

The basic geographical data, such as township boundaries, rivers, highways of the study area were provided in the form of shapefile by the State Key Laboratory of Resources and Environmental Information Systems (LREIS) of the Institute of Geographic Sciences and Natural Resources Research (IGSNRR), Chinese Academy of Sciences.

### Data sources and data process

From 2004 to 2009, 2365 CHD cases among 0- to 14-year-old children have been reported in the Sichuan Province birth defect register system of Sichuan province, including 1,224 boys and 1,141 girls, most of the cases are belonging Han Chinese. The informed consent was obtained from CHD cases’ parents or guardians. The study protocol conforms to the ethical guidelines of the 1975 Declaration of Helsinki and was approved by the Ethics Committee of the National Research Institute for Family Planning.

Each CHD case was classified and coded according to the International Classification of Diseases version 10 and belonged to the code range from Q20 to Q24.9. The classification showed that there were more than10 types of defects in our study area.

The CHD cases were distributed in 673 townships and were identified to the village level using Google Earth. Each CHD case was expressed as a point object and each point belongs to a particular township which expressed as a polygon object. The CHD cases in each township added together to calculated the CHD prevalence. The CHD cases with demographical and epidemiological information were also geocoded. Population data among the 0- to 14-year-old children every year since 2000 for each township in our study area were retrieved from the National Bureau of Statistics of China.

### HB Model

The HB model [43] is simply an extension of traditional Bayesian models in which prior distributions have some form of conditional dependence. The following simple probabilistic model was postulated. It is assumed that *O*(*i*) denotes the number of CHD cases in towhship *i* which is independent and identically Poisson distributed with the intensity parameter *λ*(*i*) = *E*(*i*) × *r*(*i*), where *E*(*i*) denotes the expected number of CHD cases in township *i*, which is proportional to the corresponding population *n*_*i*_. *r*(*i*) is the positive township-specific relative risk of CHD prevalence in township *i. O*(*i*) ~ *P*(*E*(*i*) × *r*(*i*)), *r*(*i*) is assigned a log-normal prior distribution, $\log\left[r\left(i\right)\right]\sim N\left(\mu ,\sigma_{i}^{2}\right)$, where the expectation and variance are defined by a linear function of a common value, *α*, and two independent random effects. a heterogeneous component *e*(*i*), which does not depend on the geographical location of townships and an autocorrelated component, *v*(*i*), which reflects the local spatial structure by incorporating the influence of neighboring townships. The model is

(1) $$\log\left(r\left(i\right)\right)=\alpha +\nu \left(i\right)+e\left(i\right)$$

Prior distributions are then assigned to these linear terms and consequent hyperprior distributions are assigned to the variance terms; thus, creating a hierarchical model as follows:

$$\left(\nu \left(i\right)\right|k,a,b\sim N\left(0,\kappa^{2}\right),\left(e\left(i\right)\right|\sigma ,c,d\sim N\left(0,\sigma^{2}\right),$$

$$\left(\nu \left(i\right)\right|\nu \left(j\right);j\in \mathrm{Aj}\left(i\right)\sim \mathrm{Aj}\left(\sum_{j-1}^{n}w^{*}\left(i,j\right)v\left(j\right),\;\kappa^{2}/\sum_{j-1}^{n}w\left(i,j\right)\right)$$

where *Aj*(*i*) denotes neighborhood *i*, *w(i,j)* is a weight matrix element, and w^*^(i,j) is the standardized form of the weight matrix that defines the relationship between the township *i* and its neighbor township *j*. The weight is defined simply as *w(i,j)* = 1 if two townships are adjacent(means two townships’ boundries share a common border or vertex) and *w(i,j)* = 0 otherwise.

$$1/\kappa^{2}\sim \mathrm{Gamma}\left(a,b\right),1/\sigma^{2}\sim \mathrm{Gamma}\left(c,d\right)$$

where *a* and *c* are shape parameters, *b* and *d* are inverse scale parameters. Which is the convolution Gaussian model originally proposed by Besag and Newell [44], where the random effect associated with spatial autocorrelation, *v*(*i*) is defined according to the conditional auto-regressive model(CAR) [45], the hyperprior distributions for 1/κ^2^ and 1/*σ*^2^ were specified at *Gamma*(0.5, 0.0005) in this study.

Following the Bayesian inference technique, the observed number of cases in each township was treated as a binomial random variable with parameter *P*_*i*_ in our analysis [7]. *P*_*i*_ is the probability of a live birth with CHD in township *i*. The standard prevalence is the maximum likelihood estimation of *P*_*i*_. As the environment are similar, *P*_*i*_ is assumed to be constant within the same township. *P*_*i*_ is modeled through a logit transformation, logit(*P*_*i*_), expressed as:

(2) $$\mathrm{logit}\left(P_{i}\right)=\log\left[P_{i}/1.0-P_{i}\right]=\alpha +\nu_{i}+\epsilon_{i}$$

where *α* is the intercept term(mean) used to calculate CHD prevalence, *v*_*i*_ is the spatially structured autoregression, and *ϵ*_*i*_ is the spatially unstructured random effect. A single chain sampler with number *i* of 4000 iterations were run, followed by 1000 iterations during which values were stored in the form of *P*_*i*_.

### Spatial cluster test

The first law of geography is summarized as: “Everything is related to everything else, but near things are more related than distant things” [46]. Spatial autocorrelation statistics analyzes the degree of dependency among observations in a geographical space. The fundamental goal of spatial analysis is to identify patterns in spatial data that lead to identifying a spatial autocorrelation or association and identify peculiarities in the data set in one or more regions [47].

Global spatial autocorrelation was used to test spatial correlation in the entire study area by assuming that the spatial process was the same everywhere. Spatial autocorrelation indicates that adjacent observations of the same phenomenon are correlated. Moran’s I statistic [48] is one of the most commonly used test for areal cluster analysis.

The Global Moran’s I statistic is a measure of spatial autocorrelation developed by Patrick Moran [8], the goal of which is to identify statistically significant hot-spots or clusters in data presented in spatial objects on two-dimensional surfaces. Moran’s I statistics were applied to explore the spatial clustering pattern of the birth prevalence of CHD in a quantitative way, helping researchers gain a deeper understanding of the phenomena of high CHD prevalence. The formula for global Moran’s I statistic is:

(3) $$I=\frac{n}{S_{0}}\frac{\sum_{i=1}^{n}\sum_{j=1}^{n}w_{\mathrm{ij}}\left(x_{i}-\bar{x}\right)\left(x_{j}-\bar{x}\right)}{\sum_{i=1}^{n}{\left(x_{i}-\bar{x}\right)}^{2}}$$

Where *w*(*i*,*j*) is the weight between observations *i* and *j*, and *S*_0_ is the sum of all $w_{\mathrm{ij}},S_{0}=\sum_{i=1}^{n}\sum_{j=1}^{n}w_{\mathrm{ij}}$.

The values of Moran’s Index range from -1 to +1. A Moran’s Index value near +1.0 indicates clustering, whereas an index value near -1.0 indicates dispersion. A zero values indicates a random spatial pattern, Negative (positive) values indicate negative (positive) spatial autocorrelation. In general, Moran’s I values can be transformed to Z-scores in which values >1.96 or < -1.96 indicate spatial autocorrelation significant at the 95% confidence level. The Z-score is used to evaluate the significance of the index value and is a measure of the standard deviation associated with a standard normal distribution.

Local spatial autocorrelation statistics provide estimates disaggregated to the level of spatial analysis units, allowing an assessment of the dependency relationship across space. Local clustering statistics are used to test the statistical significance of local clusters and map the extent of the clusters of the feature. The local indicators of spatial association (LISA) statistic, which is usually applied when studying local spatial clustering, is interpreted as an indicator of pockets of nonstationarity and is also used to assess the influence of individual locations on the magnitude of the global statistic as well as to identify “outliers” [39]. The LISA statistic is used to evaluate clustering of individual units by calculating the local Moran’s I statistic for each spatial unit and evaluating the statistical significance. The equation can be written as follows:

(4) $$I_{i}=\frac{Z_{i}}{m_{2}}\sum_{j=1}w_{\mathrm{ij}}Z_{j}$$

$m_{2}=\frac{\sum_{i}Z_{i}^{2}}{N},$ and $I=\sum_{i}\frac{I_{i}}{N},$ where *N* is the number of observations(units).

The Getis'sGi* statistic [40], developed by Getis and Ord, is used as a method for detecting hot-spots that measure the overall spatial association of values falling within a critical distance of each other. It can be expressed as follows:

(5) Gi*=∑jnwijd⋅γi-Wi*⋅γ¯S⋅nS1i*-Wi*2n-112

where *S* is the standard variance of CHD prevalence and *w*_*ij*_ is the spatial distance weight matrix between townships *i* and *j*. When the distance from township *j* to *i* is within distance *d*, *w*_*ij*_ (*d*) *=* 1; otherwise *w*_*ij*_ (*d*) *=* 0, and $S_{1i}^{*}$, $W_{i}^{*}$.

## Results

### CHD Prevalence mapping

Because CHD is a low probability event, the number of CHD cases among 0- to 14-year-old children from 2004 to 2009 was geocoded and aggregated by geographical units at the township level. CHD prevalence before and after adjusted by HB model among 0- to 14-year-old children were calculated.

**Figure 2:**
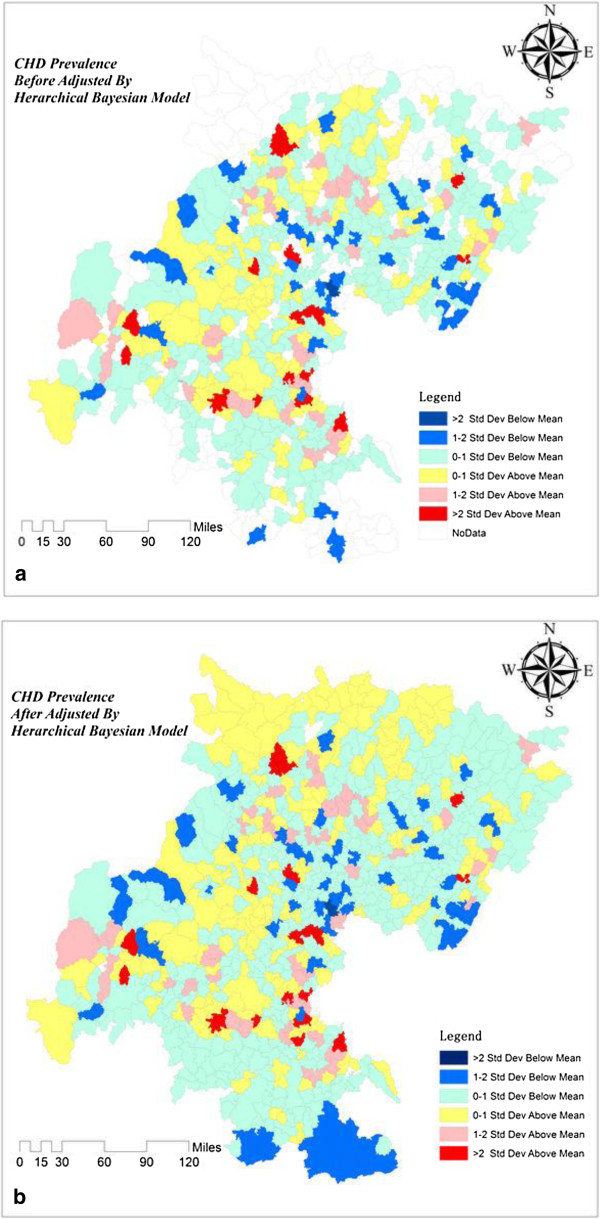
**CHD Prevalence Map Before and After Adjustment by HB. (a)** is the map of CHD prevalence before adjusted by HB model, **(b)** is the map of CHD prevalence after adjusted by HB model.

Table 1 presents the statistical characteristics of the CHD proportion estimates in the 673 townships.The prevalence Themerange map of CHD in the 673 townships was drawn and shown in Figure 2, which illustrates the distribution of CHD prevalence before adjustment by the HB model and the CHD prevalence estimates after adjustment by the HB model in the study area respectively.

**Table 1:** Prevalence of CHD before and after adjusted by HB model

**Statistics**	**0-14 year-old populations**	**CHD prevalence before-adjusted (%)**	**CHD prevalence after-adjusted (%)**
Max	263866	1.304	0.5
Min	1064	0.021	0.062
Mean	24583.89	0.018	0.170
STD	23087.47	0.013	0.058

### Spatial autocorrelation

**Figure 3:**
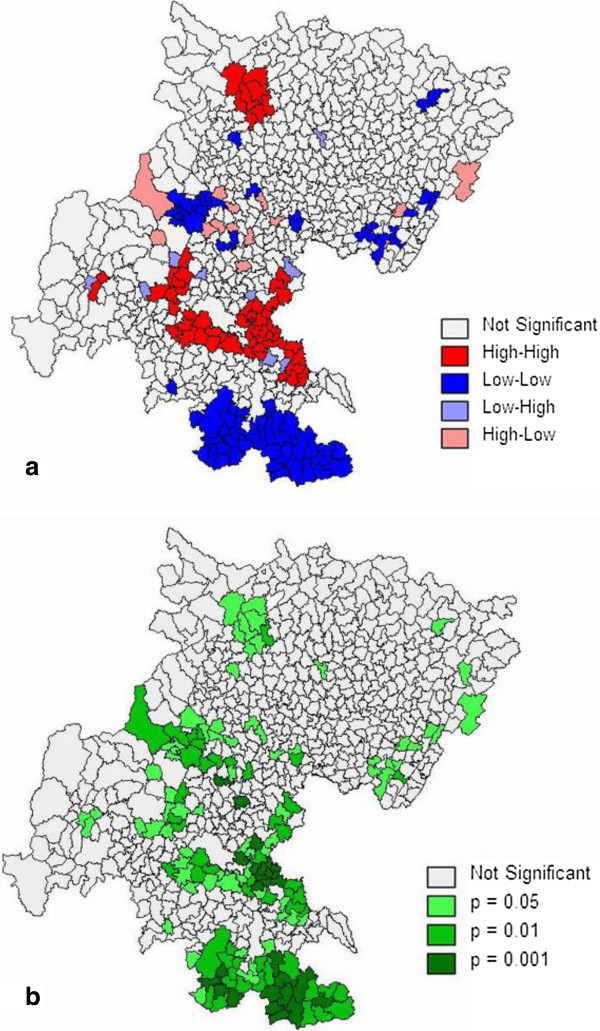
**LISA map of CHD Prevalence. (a)** is the LISA cluster map of CHD prevalence, **(b)** is the LISA significance map of CHD prevalence.

We constructed a first-order queen polygon contiguity weight matrix file of the 673 townships based on connectivity of the smallest administrative units. The global spatial autocorrelation statistic and corresponding p-values were estimated by Moran’s I statistic to HB model smoothed CHD prevalence. The computation was implemented using the Geoda0.9.5-i software. The level of spatial autocorrelation was 0.3746 (p = 0.001), suggesting non-randomness in the overall spatial pattern. The positive value indicates that the prevalence of CHD in our study area had a significant clustering pattern at the township level.The township scale LISA statistics based on the adjusted CHD prevalence using queen’s case adjacency weight matrix were calculated, and the significant clusters (HH, LL, HL, and LH) are illustrated in Figure 3.

The results of this analysis yielded to five categories of spatial units. These categories were defined as “high-high (HH),” “low-low (LL),” “high-low (HL),” “low-high (LH),” and “not significant (NS)”. The HH category indicates clustering of high adjusted CHD prevalence, whereas the LL category indicates clustering of low adjusted CHD prevalence. Three HH areas and two LL spot areas were detected. The largest HH area was detected in the central portion of NeiJiang City, and the two smaller hot-spots were found in Ya’An and MianYang. Two LL areas were observed in or just east of ChengDu city and south of LuZhou and YiBin. These outcomes were equivalent to a positive spatial autocorrelation.

In addition, the HL category indicates that high CHD prevalence values were adjacent to low values, whereas the LH category indicates that low values were adjacent to high values of adjusted CHD prevalence. These outcomes are equivalent to a negative spatial autocorrelation. Lastly, the NS category indicates that there is no statistically significant spatial autocorrelation.

### Cluster pattern

**Figure 4:**
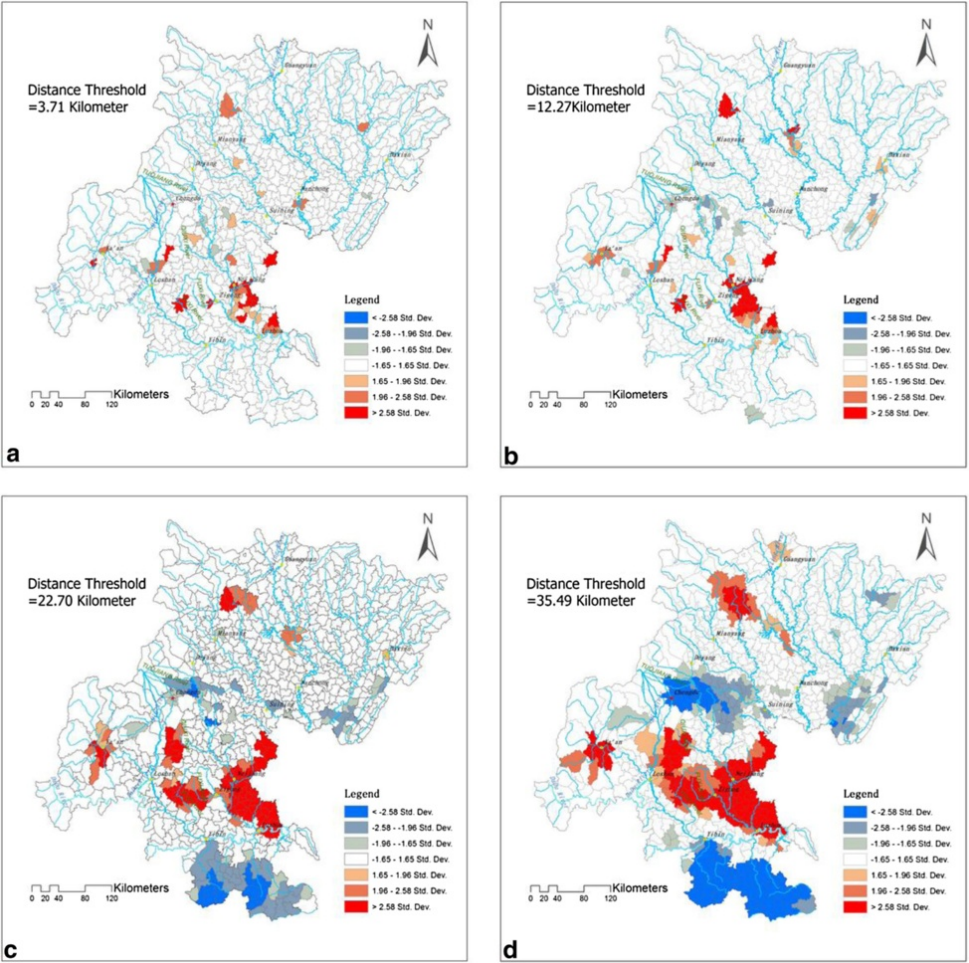
**HotspotsDetected by**Getis'sGi*. It is the hotspots map of CHD prevalence detected by Getis'sGi* statistics at different distance thresholds. **(a)** distance threshold = 3.71 km, **(b)** distance threshold = 12.27 km, **(c)** distance threshold = 22.70 km, **(d)** distance threshold = 35.49 km.

The distance value is a critical threshold of Getis'sGi* statistics. The phenomena of hot-spot distribution with different distance thresholds (The average distance between townships is 12.27 km, the shortest distance between countries is 3.71 km, the average distance between countries is 22.70 km and the shortest distance between 13 cities at prefectural level is 35.49 km in our study area) are shown in Figure 4, and the hot-spot number at different distance thresholds among 1- to 35-km is shown in Table 2.

**Table 2:** Hot/Cold spot number of CHD prevalence with different distance thresholds

	**Hot spot number**			**Cold spot number**		
**Distance threshold**	**P value < 0.01**	**0.01 < P value < 0.05**	**0.05 < P value < 0.1**	**0.1 > P value > 0.05**	**0.05 > P value > 0.01**	**P value < 0.01**
1KM	15	9	10	5	0	0
4KM	15	9	10	9	0	0
7KM	14	11	10	10	0	0
9KM	16	10	9	10	0	0
10KM	18	13	9	14	0	0
12KM	21	9	13	16	3	0
14KM	29	16	8	15	7	0
16KM	36	9	11	30	14	0
18KM	42	14	12	29	23	2
20KM	46	16	12	31	34	5
25KM	59	20	13	27	46	21
30KM	65	29	12	27	33	43
35KM	80	21	13	36	40	54

**Figure 5:**
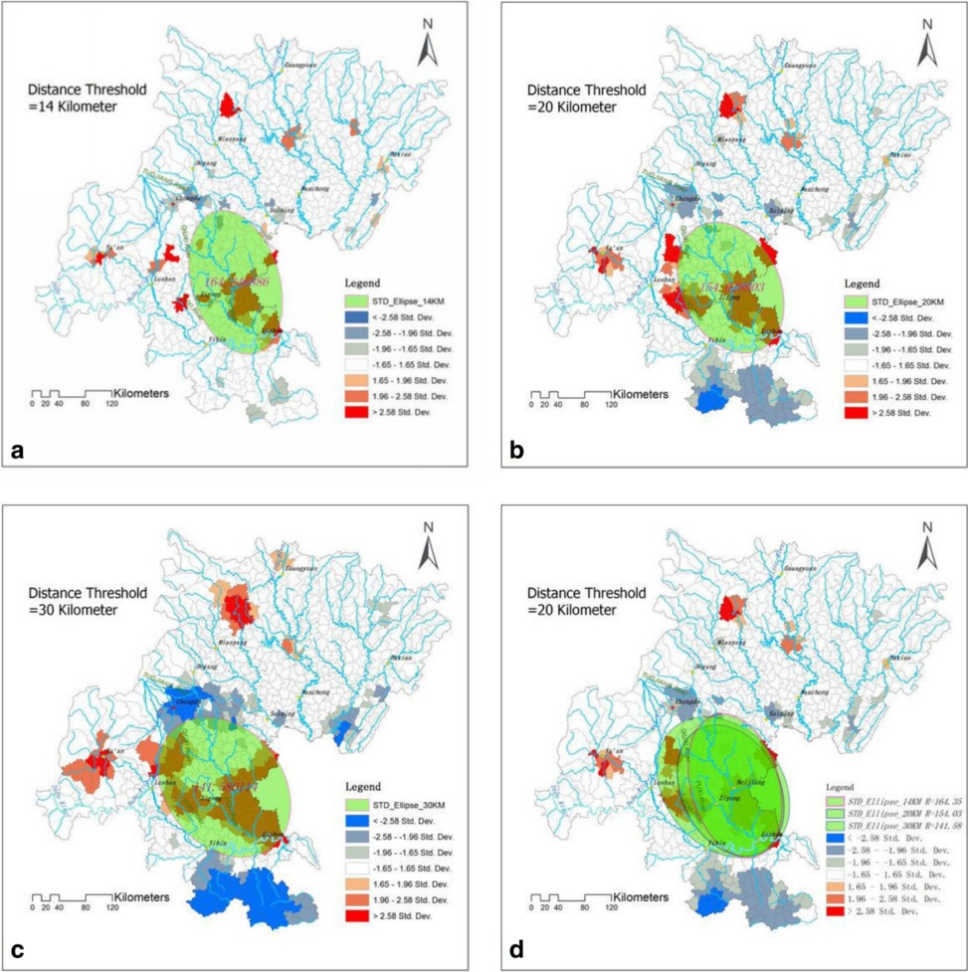
**Standard Deviation Ellipse of the CHD Prevalence Hotspot.** It is the standard deviation ellipse of the CHD prevalence hotspots detected by Getis'sGi* at different distance thresholds. **(a)** distance threshold = 14 km, **(b)** distance threshold = 20 km, **(c)** distance threshold = 30 km, **(d)** overlay of the standard deviation ellipse.

The largest hot-spot area was located on the left side of the Yangtze River and downstreams of the Tuojiang River watershed, including the NeiJiang, ZiGong, MeiShan, and LeShan areas. One of the cold-spot areas was located on the right side of the Yangtze River and was symmetrically distributed with the largest hot-spot area. Within the distance from 1 km to 9 km, the variations in the hot-spot cluster did not change. The hot-spot cluster’s aggregate began at the distance threshold value of 10 km. The center of the hot-spot area was located in NeiJiang city, and the standard deviation ellipse statistics showed that the hot-spot region extended along the flow direction of the TuoJiang River and was present as a zonal distribution trend, which is shown in Figure 5.

## Discussion

CHD is the most frequent group of congenital anomalies and is the leading cause of infant death due to congenital anomalies and is associated with a considerable burden on public and private resources [13].

In this study, the HB model was used to adjust the prevalence of CHD. The geographical distribution of CHD prevalence at the township level was investigated and mapped, both global and local spatial clustering methods were used to quantify the spatial pattern of CHD prevalence. Moran’s I statistic was used as a measure of global clustering and was assessed by testing the null hypothesis that the spatial pattern of these data were random. LISA is an indicator of local spatial association that measures whether CHD prevalence for a particular spatial unit at the township scale is closer to the values of a neighboring unit or to the average of the study area.

We found significant spatial variability in the prevalence of CHD in 0- to 14-year-old children in Sichuan Basin. In addition, the significant positive spatial autocorrelation and the significant local clusters confirmed the spatial variances of CHD prevalence. The spatial pattern and clustering of events provide important information for developing and refining geographical-and population-specific prevention programs to reduce CHD risk. In addition, this information will be useful to healthy planners because many current policies and health initiatives are principally based on assumptions of spatial homogeneity.

### Explanatory phenomena

**Figure 6:**
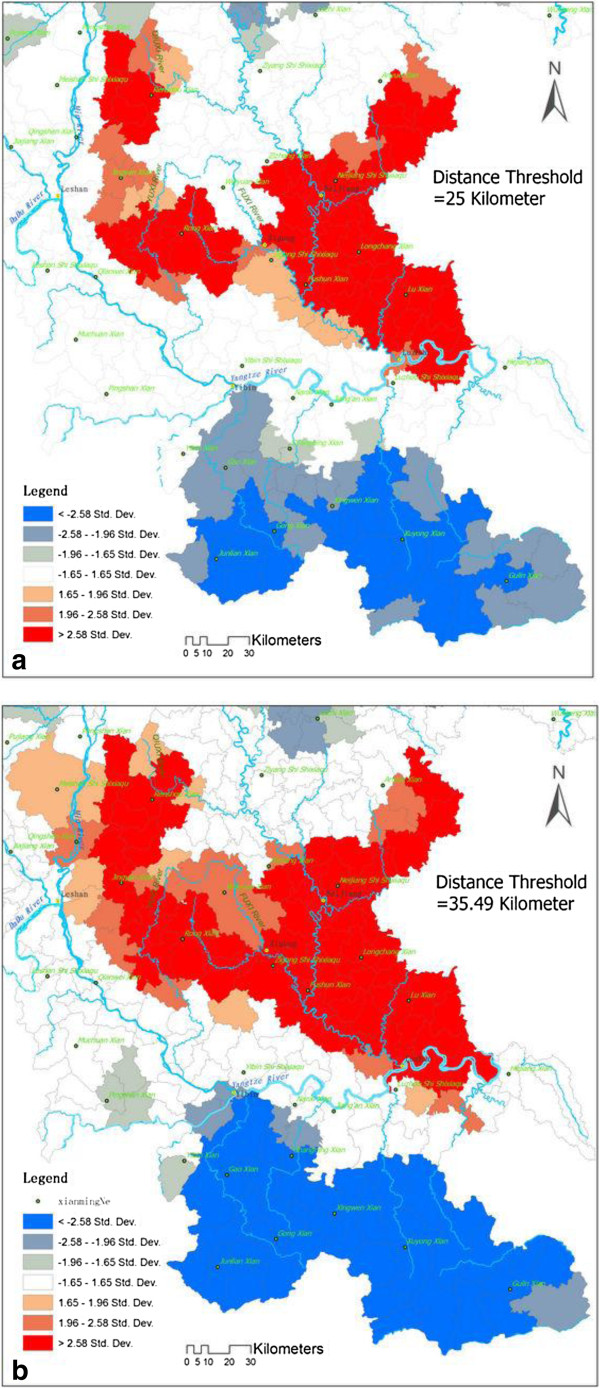
**Hot-Spot of CHD Prevalence in TuoJiang River Watershed. (a)** Hot-Spot map where distance threshold = 25 km, **(b)** Hot-Spot map where distance threshold = 35.49 km.

Identifying CHD clusters provides clues to causality. The largest CHD hot-spot region was located in the TuoJiang River watershed, as shown in Figure 6. Thus, a stronger relationship was observed between CHD prevalence and the environment around the TuoJiang River.

The Tuojiang River is one of the largest tributaries of the upper Yangtze River, industries and agriculture are well developed along the coastal area of the TuoJiang River. Pollution from chemical plants, machinery and paper industries as well as non-point source pollution from rural regions is a very serious issue. A literature review showed that heavy metal pollution in sediments increases from up to downstream of the Tuojiang River. Mining activities are the most important sources of heavy metals, and heavy metal contents clearly increase at the convergence region of the Tuojiang River. The potential ecological risk from cadmium is the highest, followed by that of arsenic and lead [49,50]. In addition, the average total nitrogen and total phosphorus concentrations in the Tuojiang River exceed the standard acceptable value by more than 3-and 1.2-folds, respectively [51,52]. All of these characteristics are potential risk factors for a high prevalence of CHD.

### Limitations

Three major limitations of this study should be discussed. First, the calculation of CHD prevalence was a key step in the study. The reported prevalence of CHD at birth varies widely worldwide. In our study, the newborn rate in Sichuan Province was 8.93% in 2010, which was cited from the Sixth National Population Census from the National Bureau of Statistics, China. The prevalence of CHD in our study area was lower than this value because we only considered surviving children with CHD in each family as per the current family planning policy in China.

The second limitation was that spatial patterns of CHD prevalence may change dependence on the spatial scales and units used in analysis, which is commonly known as a modifiable areal unit problem or ecological fallacy [53]. The importance of location, spatial interaction, spatial structure, and spatial processes has been well established in public health literature. The utility of exploratory spatial data analysis tools allows researchers to map spatial patterns, identify local variability in CHD prevalence, and assess the efficacy of spatial models. The objectives of this study were to help generate working hypotheses and design a more sophisticated research protocol for future research efforts. Studying different distributions and spatial patterns (point or lattice) at different spatial scales (country or village level) deserves further research.

The third limitation was CHD defects include abnormal chromosomes, single-gene disorders, and polygenic disorders. But the prevalence of CHD differs in different areas within a limited region. Geographical variations in CHD prevalence can be explained by variations in socioeconomic status, education, urbanization, climatological factors, ethnicity, and patient-related factors such as comorbidity, lifestyle, and healthcare-seeking behavior. More insight into the epidemiology of CHD is needed. Exploring the environmental risk factors for CHD is also a difficult problem. Maternal factors, maternal health, and diseases such as diabetes mellitus, phenylketonuria, febrile illness,rubella,stress, and obesity have significant relationship with CHD. Maternal lifestyle, drug and medical use, and environmental toxic exposure lead to CHD.

## Conclusions

It is very intriguing that the high prevalence of CHD was associated with watershed environmental pollution and specific environmental factors in specific areas. Potential risk factors contribute to CHD, and the mechanism of the environmental risk factor effects deserves special attention. In addition, considering more potential risk factors from the epidemiology perspective and applying different spatial statistical methods are important strategies in CHD studies.

Exploring the spatial and temporal changes in CHD prevalence, reducing the recurrence of CHD, and preparing prevention strategies are new challenges for subsequent studies. We hope that information is gleaned from this study and that more in-depth studies are based on this research. Identifying causal agents of CHD using geographical analysis technology and tools to provide public health professionals and policy makers within areas of elevated risk are important for designing effective intervention programs.

## Competing interests

The authors declare they have no competing financial or non-financial interests.

## Authors' contributions

L-GM contributed to the initial study design, data acquisition, data processing and analysis, interpretation of results and drafting of the maps and the manuscript. All authors have read and approved the final manuscript.

## Pre-publication history

The pre-publication history for this paper can be accessed here:

http://www.biomedcentral.com/1471-2458/14/595/prepub

## References

[B1] van der Linde D Konings EEM Slager MA Witsenburg M Helbing WA Takkenberg JJM Roos-Hesselink JW Birth prevalence of congenital heart disease WorldwideA systematic review and meta-analysis J Am Coll Cardiol 2011 58 21 2241 2247 10.1016/j.jacc.2011.08.025 22078432

[B2] Bernier PL Stefanescu A Samoukovic G Tchervenkov CI The challenge of congenital heart disease worldwide: epidemiologic and demographic facts Semin Thorac Cardiovasc Surg Pediatr Card Surg Annu 2010 13 26 34 10.1053/j.pcsu.2010.02.005 20307858

[B3] Liu S Liu J Tang J Ji J Chen J Liu C Environmental risk factors for congenital heart disease in the Shandong Peninsula, China: a hospital-based case–control study J Epidemiol 2009 19 3 122 130 10.2188/jea.JE20080039 19398851 PMC3924136

[B4] Liu YL The advancement and challenges of management of infant and young children's congenital heart defect in China Nat Med J China 2004 84 11 881 884 15329268

[B5] Ouyang N Luo J Du Q Liu Z Case–control study on environmental factors in congenital heart disease J Cent S Univ Med Sci 2011 36 2 159 164 10.3969/j.issn.1672-7347.2011.02.012 21368427

[B6] Zhang Y Riehle-Colarusso T Correa A Li S Feng X Gindler J Lin H Webb C Li W Trines J Berry RJ Yeung L Luo Y Jiang M Chen H Sun X Li Z Observed prevalence of congenital heart defects from a surveillance study in China J Ultrasound Med 2011 30 7 989 995 21705732 10.7863/jum.2011.30.7.989 PMC4469985

[B7] Wu J Wang J Meng B Chen G Pang L Song X Zhang K Zhang T Zheng X Exploratory spatial data analysis for the identification of risk factors to birth defects BMC Public Health 2004 4 1 23 10.1186/1471-2458-4-23 15202947 PMC441386

[B8] Mocumbi AO Lameira E Yaksh A Paul L Ferreira MB Sidi D Challenges on the management of congenital heart disease in developing countries Int J Cardiol 2011 148 3 285 288 10.1016/j.ijcard.2009.11.006 19932516

[B9] Li H Calder CA Cressie N Beyond Moran's I: testing for spatial dependence based on the spatial autoregressive model Geogr Anal 2007 39 4 357 375 10.1111/j.1538-4632.2007.00708.x

[B10] Klemetti A Environmental factors and congenital malformations, a prospective study Acta Ophthalmol (Copenh) 1968 46 3 350 351 5755728 10.1111/j.1755-3768.1968.tb02814.x

[B11] Nora JJ Multifactorial inheritance hypothesis for the etiology of congenital heart diseases: the genetic-environmental interaction Circulation 1968 38 3 604 617 10.1161/01.CIR.38.3.604 4876982

[B12] Brennan P Young ID Congenital heart malformations: aetiology and associations Semin Neonatol 2001 6 1 17 25 10.1053/siny.2000.0032 11162282

[B13] Blue GM Kirk EP Sholler GF Harvey RP Winlaw DS Congenital heart disease: current knowledge about causes and inheritance Med J Aust 2012 197 3 155 159 10.5694/mja12.10811 22860792

[B14] Dolk H Epidemiologic approaches to identifying environmental causes of birth defects Am J Med Genet C: Semin Med Genet 2004 125C 1 4 11 10.1002/ajmg.c.30000 14755428

[B15] Armstrong BG Dolk H Pattenden S Vrijheid M Loane M Rankin J Dunn CE Grundy C Abramsky L Boyd PA Stone D Wellesley D Geographic variation and localised clustering of congenital anomalies in Great Britain Emerg Themes Epidemiol 2007 4 14 10.1186/1742-7622-4-14 17617898 PMC1939702

[B16] Greer W Sandridge AL Al-Menieir M Al Rowais A Geographical distribution of congenital heart defects in Saudi Arabia Ann Saudi Med 2005 25 1 63 69 15822500 10.5144/0256-4947.2005.63 PMC6150559

[B17] Cronk CE Gangnon R Cossette S McElroy JA Pelech AN Modeling geographic risk of complex congenital heart defects in Eastern Wisconsin Birth Defects Res Part A: Clin Mol Teratol 2011 91 7 631 641 10.1002/bdra.20828 21630424

[B18] Cavero Carbonell C Zurriaga O Pérez Panadés J Barona Vilar C Martos Jiménez C Temporal variation and geographical distribution: congenital heart defects in the Comunitat Valenciana Anales de Pediatria (Barcelona, Spain: 2003) 2013 79 3 149 156 10.1016/j.anpedi.2012.12.007 23481464

[B19] Agay-Shay K Amitai Y Peretz C Linn S Friger M Peled A Exploratory spatial data analysis of congenital malformations (CM) in israel, 2000–2006 ISPRS Int J Geo-Inform 2013 2 1 237 255 10.3390/ijgi2010237

[B20] Miao CY Li WX Geng D Tao LA Zuberbuhler JS Zuberbuhler JR Effect of high altitude on prevalence of congenital heart disease Chin Med J 1988 101 6 415 418 3146471

[B21] Sandridge AL Greer W Al-Menieir M Al Rowais A Exploring the impact of altitude on congenital heart defects in Saudi Arabia Avicenna 2010 2010 3

[B22] Miao CY Zuberbuhler JS Zuberbuhler JR Prevalence of congenital cardiac anomalies at high altitude J Am Coll Cardiol 1988 12 1 224 228 10.1016/0735-1097(88)90378-6 3379209

[B23] Davies BR The seasonal conception of lethal congenital malformations Arch Med Res 2000 31 6 589 591 10.1016/S0188-4409(00)00245-9 11257326

[B24] Samanek M Slavik Z Krejcir M Seasonal differences in the incidence of congenital heart defects Czech Med 1991 14 3 146 155 1807932

[B25] Sandahl B Seasonal incidence of some congenital malformations in the central nervous system in Sweden, 1965–1972 Acta Paediatr Scand 1977 66 1 65 72 10.1111/j.1651-2227.1977.tb07809.x 318790

[B26] Valadez A Meltzer AA Seasonal variation in the incidence of congenital malformations in San Miguel de Allende, Mexico Prog Clin Biol Res 1990 341A 741 745 2217293

[B27] Grech V Seasonality in live births with congenital heart disease in Malta Cardiol Young 1999 9 4 396 401 10476830 10.1017/s1047951100005205

[B28] Dadvand P Rankin J Rushton S Pless-Mulloli T Ambient air pollution and congenital heart disease: A register-based study Environ Res 2011 111 3 435 441 10.1016/j.envres.2011.01.022 21329916

[B29] Dolk H Armstrong B Lachowycz K Vrijheid M Rankin J Abramsky L Boyd PA Wellesley D Ambient air pollution and risk of congenital anomalies in England, 1991–1999 Occup Environ Med 2010 67 4 223 227 10.1136/oem.2009.045997 19819865

[B30] Dadvand P Rankin J Rushton S Pless-Mulloli T Association between maternal exposure to ambient Air pollution and congenital heart disease: a register-based spatiotemporal analysis Am J Epidemiol 2010 173 2 171 182 21123851 10.1093/aje/kwq342 PMC3011953

[B31] Rankin J Chadwick T Natarajan M Howel D Pearce MS Pless-Mulloli T Maternal exposure to ambient air pollutants and risk of congenital anomalies Environ Res 2009 109 2 181 187 10.1016/j.envres.2008.11.007 19135190

[B32] Agay-Shay K Friger M Linn S Peled A Amitai Y Peretz C Air pollution and congenital heart defects Environ Res 2013 124 28 34 23623715 10.1016/j.envres.2013.03.005

[B33] Vrijheid M Martinez D Manzanares S Dadvand P Schembari A Rankin J Nieuwenhuijsen M Ambient air pollution and risk of congenital anomalies: a systematic review and meta-analysis Environ Health Perspect 2011 119 5 598 21131253 10.1289/ehp.1002946 PMC3094408

[B34] Goldberg SJ Lebowitz MD Graver EJ Hicks S An association of human congenital cardiac malformations and drinking water contaminants J Am Coll Cardiol 1990 16 1 155 164 10.1016/0735-1097(90)90473-3 2358589

[B35] Dolk H Vrijheid M The impact of environmental pollution on congenital anomalies Br Med Bull 2003 68 25 45 10.1093/bmb/ldg024 14757708

[B36] Kuciene R Dulskiene V Selected environmental risk factors and congenital heart defects Medicina (Kaunas) 2008 44 827 832 19124958

[B37] Stoupel E Birk E Kogan A Klinger G Abramson E Israelevich P Sulkes J Linder N Congenital heart disease: correlation with fluctuations in cosmophysical activity, 1995–2005 Int J Cardiol 2009 135 2 207 210 10.1016/j.ijcard.2008.03.053 18582962

[B38] Stoupel EDV Kuciene R Abramson E Israelevich P Sulkes J Congenital heart disease (CHD) and environmental physical activity, kaunas, 1995–2005 Sun and Geosphere 2009 4 2 45 49

[B39] Anselin Local indicators of spatial association: LISA Geogr Anal 1995 27 3 93 115

[B40] Getis A Ord JK The analysis of spatial association by use of distance statistics Geogr Anal 1992 24 3 189 206

[B41] Spiegelhalter D Thomas A Best N Lunn D WinBug program version 1.4 Biostatistics Unit, Cambridge 2003 7 8 9

[B42] Sichuan http://en.wikipedia.org/wiki/Sichuan

[B43] Haining RP Spatial Data Analysis: Theory and Practice 2003 Cambridge: Cambridge University Press

[B44] Besag J Newell J The detection of clusters in rare diseases J R Stat Soc Ser A Stat Soc 1991 154 143 155 10.2307/2982708

[B45] Besag J Spatial interaction and the statistical analysis of lattice systems J R Stat Soc Ser B Methodol 1974 36 2 192 236

[B46] Tobler WR A computer movie simulating urban growth in the Detroit region Econ Geogr 1970 46 234 240

[B47] Ord JK Getis A Testing for local spatial autocorrelation in the presence of global autocorrelation J Reg Sci 2001 41 3 411 432 10.1111/0022-4146.00224

[B48] Moran PA Notes on continuous stochastic phenomena Biometrika 1950 37 1–2 17 23 15420245

[B49] Jia-xuan L Ze-ming S Lin Z Shi-jun N Evaluation on potential ecological risk of heavy metals pollution in sediments from Tuojiang drainage Earth Environ 2010 4 017

[B50] TingTing W Vertical Distributions of Various Bacteria and Arsenic Species in Sediments of Tuojiang River in Different Seasons 2008 PhD Thesis,Chengdu: University of Technology, Analytical Chemistry

[B51] Jian-ping L Study of water quality analysis and pollution status of Tuojiang river in the area of Fushun Sichuan Environ 2013 32 2 23 26

[B52] WU Y Deng T XU Q Guo Y Environmental pollution behaviors of Pb and Cd in fluvial sediments in Tuojiang river Guangdong Trace Ele Sci 2010 17 9 22 28

[B53] Hayward PM The Modifiable Areal Unit Problem (MAUP) and Health Disparities 2009 Proquest: Umi Dissertation Publishing

